# An On-Film AMC Antenna Insert-Molded in Earbuds with Enhancement in In-Ear and In Situ Received-Signal Sensing

**DOI:** 10.3390/s22124523

**Published:** 2022-06-15

**Authors:** Yejune Seo, Inyeol Moon, Junghyun Cho, Yejin Lee, Jiyeon Jang, Morimoto Shohei, Kurosaki Toshifumi, Sungtek Kahng

**Affiliations:** 1 Department of Information & Telecommunication Engineering, Incheon National University, Incheon 22012, Korea; m.june@inu.ac.kr (Y.S.); iy-moon@nissha.com (I.M.); elsa@inu.ac.kr (J.C.); allen@inu.ac.kr (Y.L.); yeon.jj@inu.ac.kr (J.J.); 2 Global R&D Center, NISSHA Korea, Inc., 7F, 26, Hwangsaeul-ro 312beon-gil, Bundang-gu, Seongnam-si 13591, Gyeonggi-do, Korea; 3 NISSHA Co., Ltd., 3 Mibu Hanai-Cho, Nakakyo-ku, Kyoto 604-8551, Japan; sh-morimoto@nissha.com (M.S.); ts-kurosaki@nissha.com (K.T.)

**Keywords:** earphone, wearable antenna, AMC antenna, metamaterial structure, received signal strength sensing, head–ear phantom

## Abstract

In this paper, a novel thin and flexible antenna is proposed for earbuds to gain an improvement in their wireless signal-sensing capability as a film-based artificial magnetic conductor (AMC) structure. As antenna designs for earbuds face challenges of being embedded beneath the top cover of the earbud, conformal to curved surfaces, and very close to metallic ground and touch-panel parts, as well as scarce degrees of freedom from feeding conditions and functional degradation by human tissue, unlike conventional techniques such as quasi quarter-wavelength radiators on LDS and epoxy molding compounds (relatively thick and pricy), an antenna of a metal pattern on a film is made with another film layer as the AMC to mitigate problems of the antenna in a small and curved space of an insert-molded wireless device. The antenna was designed, fabricated, and embedded in earbud mockups to work for the 2.4 GHz Bluetooth RF link, and its functions were verified by RF and antenna measurement, showing that it could overcome the limitations in impedance matching with only lumped elements and poor radiation by the ordinary schemes. The input reflection coefficient and antenna efficiency were 10 dB and 9% better than other methods. In particular, the on-film AMC antenna (OFAA) presents robustness against deterioration by the human tissue, when it is placed in the ear phantom at the workbench and implemented in an in situ test using a large zorb ball mimicking a realistic sensing environment. This yielded an RSSI enhancement of 20–30 dB.

## 1. Introduction

People wear smartwatches and can check their heartbeats while walking or running. Wearing earbuds, they listen to music wirelessly streamed from their phones. Home appliances are being advertised to perform eye-capturing jobs under the name of smart fridges or smart TVs. These e-Health monitoring, entertainment, and home electronics have smart as the prefix. These subjects are handled in the area of Internet of things (IoT) [1].

IoT mixes the strengths of electronics and networking. Sensors and communication modules are distributed in a network and across multiple networks [2,3,4,5,6,7,8]. Software in the IoT equipment watches and controls the data traffic, resource allocation, and choice of a network and conversion to different routes. This dynamic networking is operated wirelessly, which necessitates a contribution from RF technologies as part of electronics for IoT. Looking at what is required in RFID, antennas for tags and the reader in the service play crucial roles. In particular, when tags are laid in an unfriendly environment such as lossy objects or shadows, simple antenna design techniques and the use of commercial antennas are inadequate for wireless links of a reasonable quality.

As the RFID technology revealed its limitations, it has pushed wireless service developers to suggest M2M (machine-to-machine), D2D (device-to-device), IoE (Internet of everything) to reach the era of IoT (Internet of things). Although the previous and current mottos of wireless connectivity do not seem to overlap, there are things to solve in common. Time-varying conditions and signal strength between multiple nodes in a network should be considered and handled seriously. Among a number of approaches to satisfactory communication from the TX-node to the RX-node, advanced antennas play an important role in coping with the problem. Thus, it is worthwhile to review antennas used in sensor networking, BLE services, and wireless body-area networking.

Almost all the antennas aimed at IoT applications are basically small. The survey of the related literature shows that many antennas are commonly made on a PCB without housing, which is ideal. In contrast, commercial products need enclosure. While some antennas are in harsh environmental conditions such as being near human tissue or having an obstacle in the line of sight, others are not. A circular patch monopole was introduced for body-centric communication by Sun et al. [9]. The antenna could be realized on textiles and was tested with bending and crumpling. One presented a dual-mode property to a microstrip by having a meander line inside a loop [10]. The ground plane exists under the two radiating lines to prevent unwanted coupling from a human tissue-mimicking cylinder. Liu et al. etched the metal ground of a microstrip ring antenna similar to parallel rings to generate a dual-band resonance [11]. Despite indicating body-centric communication, the human phantom test was not performed. The top of an antenna was separated from the bottom through the air gap, and there were relatively long vias between the two layers for dual-band on- and off-body communication [12]. It was taller than other wearable antennas and was adopted to a button of shirts worn by a human being. Because of the adequate height to the antenna from the ground, its performance was not disturbed by the examinee. According to Atash et al., a triangular monopole for a dual-band operation can be located over periodic patches to minimize the negative effects from the human body [13]. Its characteristics were checked while placed on the human abdomen. A pork slab was used for testing by T. Shaw et al. to verify the functions of a circular patch laid over an array of rings [14]. A slotted circular patch antenna was worn by a person to determine the frequency variation for on-body/off-body communication [15]. PIN diodes were loaded to change the state. Arif et al. used a slotted metal ground for a fractal-shaped patch on top for WBAN communication. They observed the effects of human tissue on the antenna performance by attaching it to a person [16]. Having on-body communication in mind, Li et al. used two wide planes to feed a bowtie dipole supported by a partially meandered loop [17]. They tested their antenna without a human phantom but with a PEG–water solution. Instead of liquid material, an electromagnetic model of the human body was applied using a tri-band antenna made up of a stub dipole and a slot dipole on a large CPW (coplanar-waveguide) ground [18]. Their antenna was made with RT 5880 as the substrate, but polytetrafluoroethylene (PTFE) was adopted in ink-jet printing for other body-centric communication cases [19]. Su et al. printed a cluster of mushrooms on a PTFE top layer, used in the mode-tracking of human activity recognition. This device could not handle the RF link whose path included the head.

In addition to the work mentioned above, research has considered the head for special requirements. A voxel head was considered for the attachment of a cross-bowtie antenna [20]. Vaezi et al. generated a circular polarization from the antenna on an FSS. Al-Adhami et al. formed 3 × 2 Minkowski fractals on a patch mounted on the very top of the human head [21]. The multi-wound slots caused the antenna to resonate at 403 MHz, away from the BLE service. A dual meandered loop antenna was replicated in a chain of sensors wrapping the head to detect the presence of liquid inclusion in the head [22]. Alqadami et al. mimicked the monitoring of brain stroke. Cihangir et al. presented a patch-coupled monopole and a loop antenna separately on two ends of the long side of a goggle [23]. This approach did not work on earphones. They put the wearable device around a specific anthropomorphic mannequin (SAM) and observed the radiated field patterns. Many researchers have addressed body-centric connectivity using phones, goggles, watches, and tags on conductive textiles, but few have handled antennas for headsets or earphones, supposing that they are relatively thin and small. An ear-set was spotted in the title of Zahid et al.’s work, where they took advantage of speaker wires for their antenna [24]. Their antenna necessitated its own PCB for feeding and grounding, forcing its existence outside of the ear-set, which is not appealing for realistic prototyping. Zhekov wrote a comprehensive review on the electromagnetic characteristics of a variety of commercial earphones [25]. Although they did not make an attempt to design antennas for the devices, they conducted interesting experiments on the EIRP (effective isotropic radiated power) of each of the products, as well as the electric field intensity, when a volunteer wore the DUTs. The SAM was not used, but the fact that in-the-ear wearable devices were brought is worthy of attention.

In this paper, a body-centric communication device fit into the ear was realized with a standard SAM to emulate the real use of earbuds. More concretely, an on-film AMC antenna (OFAA) as a small and flexible radiating element was devised and put into a mock-up earbud. This differs from other metamaterials [26,27,28,29,30,31,32]. While it is not an AMC and is not fit for earbuds, this novel structure was placed into an ear phantom attached to a head SAM, representing the most advanced model. The antenna is differentiated from other antennas in that it consists of a shorter radiating element on the upper film layer and an AMC on the lower film layer to overcome challenges in the impedance matching and radiation properties of an antenna insert-molded in an extremely small space in the vicinity of the ground and touch panel as metal parts under a dielectric cover of *ε*_r_ = 3.2. Owing to the film-type AMC, with just a 0.55 mm gap, the radiator whose length was one-twelfth of the wavelength obtained a good impedance match and an antenna efficiency higher than that of the quarter-wavelength case. The SAR was reduced, meeting the standard. These merits became more commendable in further steps where the OFAA-embedded earbuds were placed in ear phantoms, and their RF and communication link evaluation actions were taken at the workbench and using a zorb ball in situ test setup. To evaluate the Bluetooth signal transmission and reception, a plastic ball called a zorb with a diameter of 3 m was made, and the in situ wireless link was examined using the ear-phantom-mounted earbuds located at the center of the zorb ball, whose outer surface had a grid with nodes holding the receiving sensor as part of the RSSI chipset. The series of tests presented that the proposed antenna design method enhanced the impedance matching and electromagnetic radiation performance with aggravating conditions during feeding and mounting, and it was robust against undesirable effects from the human tissue. The input reflection coefficient and antenna efficiency were improved by 10 dB and 9% compared to the conventional antenna. The theoretical and experimental aspects of the proposed design and test scheme are discussed from the viewpoint of antenna design and its performance in the next section, while its integration into earphone mock-ups and its frequency responses to the RSSI test using the head–ear phantom and the zorb ball setting are presented in the last section.

## 2. Design of the On-Film AMC Antenna and Its Performance

### 2.1. Basic Aspects of the Earbud Antenna Design

**Figure 1:**
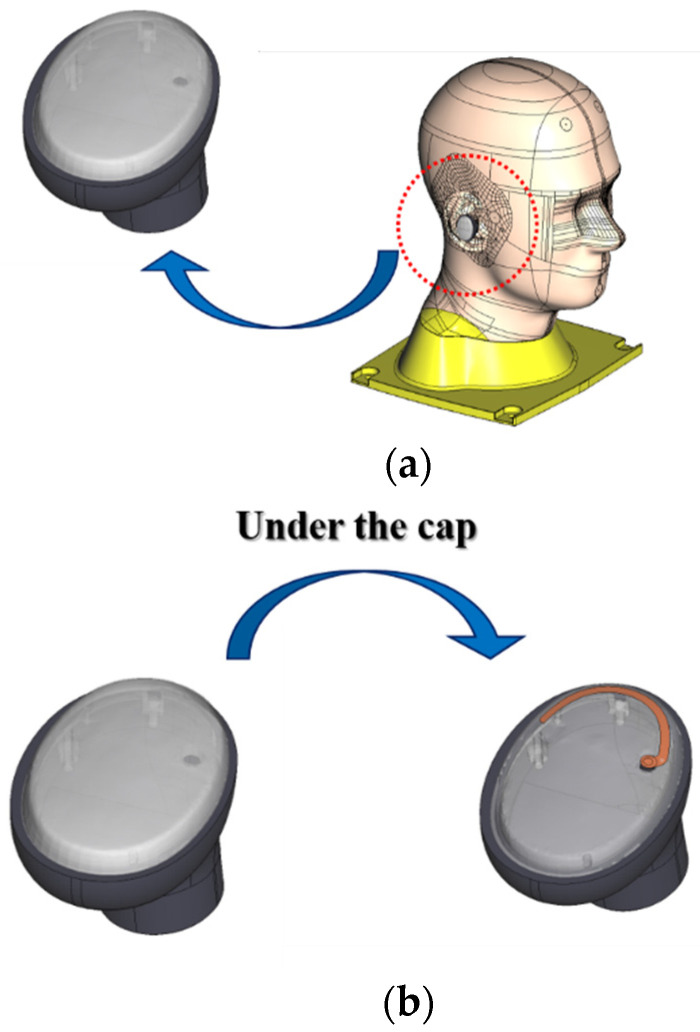
The earphone placed in the ear of the SAM: (**a**) overall view; (**b**) antenna in the earbud.

Obviously different from mobile handsets or smart glasses, an earphone faces more challenging conditions in design. It is surrounded by human tissue, and a really small space is provided for antenna placement, as shown in Figure 1.

Figure 1a shows how the earphone sits on the head phantom. While the human tissue is shown under the mobile handheld antenna, regarding the present work, there was human tissue around the earphone. This means that the antenna for the earphone would likely be disturbed by the human tissue with a much higher dielectric constant and loss tangent. This drawback should be overcome. The design procedure considered the latest head–ear model, which copies the real human head and ears under the IEC standard, including the ear meatus or canal and acoustic auricle or tragus/anti-tragus, concha, and helix of the human ear. Accordingly, POPEYE-V10 was procured along with its computer-aided design model, unlike other phantoms which do not possess ear canals or the concha. A popular earbud was chosen and is depicted in Figure 1b. There are diverse shapes and sizes, and a short and compact device was selected. While others used a stick with a helical antenna or chip antenna, the earbud chosen did not allow long and straight or thick antennas. Inside the dielectric cover of the earbud, many parts and layers were present. The majority of the internal space was taken up by electronic parts of the touch panel and wire grid, the speaker, the battery, the control and signal processing chip, and the interconnects and fixtures. A really small and shallow space was allowed for the antenna, including the impedance matching. Others resorted to monopole antennas or PIFAs for the earphone under discussion [15]. This does not allow overcoming the problems of poor impedance matching and weak radiation posed by the internal configuration of the earbud. Hence, a novel approach is required; in this study, an antenna was made on a thin film insertable to the small space and backed up by an on-film AMC to increase the radiation and maintain the good antenna performance in the ear phantom. The design of the OFAA proceeded with the conventional antenna on the film as a comparative study, highlighting the advantages of the proposed method.

### 2.2. Proposed Approach of Novel Earbud Antenna

**Figure 2:**
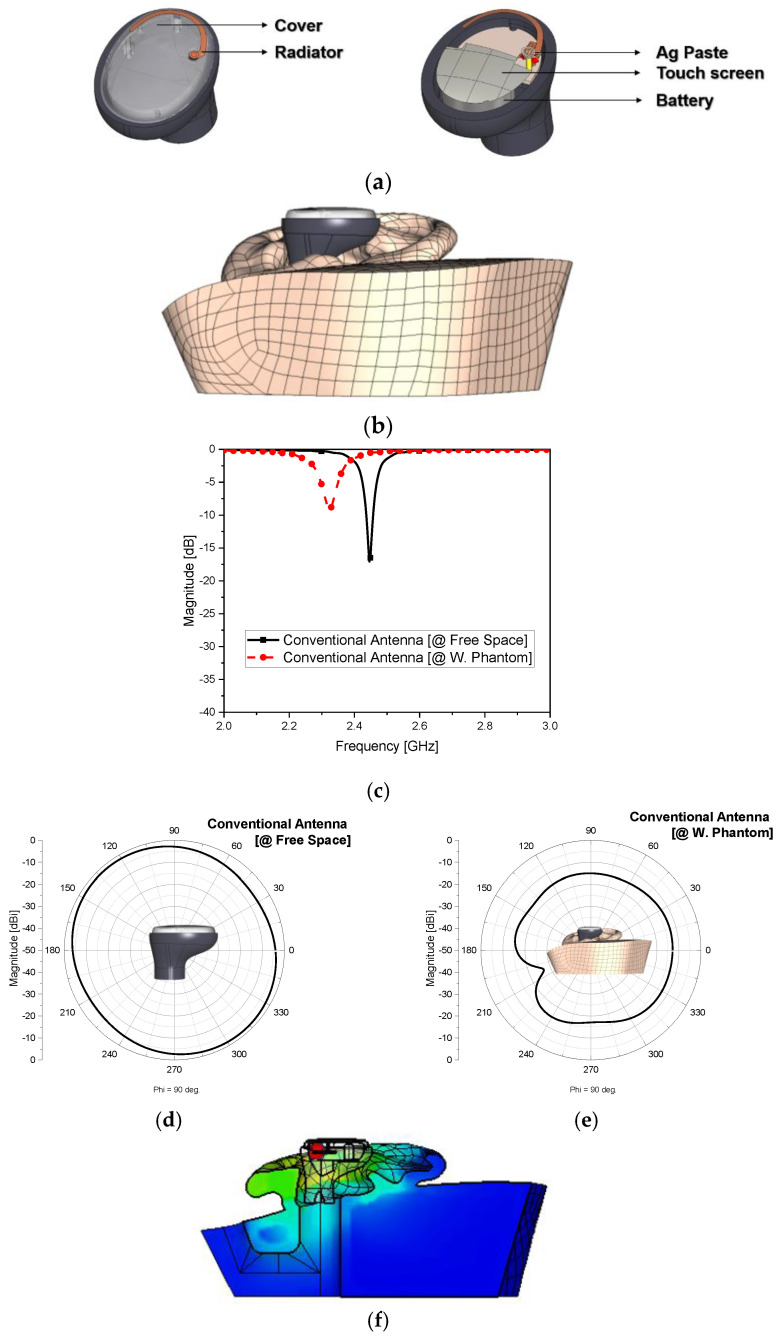
Investigating the conventional antenna: (**a**) geometry; (**b**) attached to the human phantom; (**c**) S_11_; (**d**) radiated field pattern of the modified monopole antenna in the free space; (**e**) radiated field pattern of the modified monopole antenna with the phantom; (**f**) SAR.

As shown in Figure 2a, an antenna as a modified monopole whose size was almost one-quarter of the wavelength was formed on the top film layer in LDS and epoxy molding compounds (bulky and long), tapping the circular disc of the vertical conductor from the feeding circuit. The vertical metal was a pogo-pin bridging the gap between the radiating element layer (top) and feeding circuit layer (bottom). The joints of this 3D structure involving the chain of a flat plane (feed-circuit), a vertical column (3 mm long pogo-pin), and a curved surface (antenna radiator) caused impedance mismatch and exacerbated radiation. An L-network of a chip inductor and a chip capacitor is needed in the feeding circuit to tackle these problems. Using 1.6 nH and 2.0 pF for the lumped elements L and C on a 15 mm × 11.5 mm PCB, the antenna in the free space was tuned to 2.45 GHz, as shown in Figure 2b; however, it was shifted by 120 MHz and worsened by 7.9 dB, as shown in Figure 2c, upon being mounted on the human tissue. At the same time, the far-field pattern in Figure 2d,e was obtained with an antenna efficiency of 53%. It is helpful to keep the equations below in mind when describing the relationships between the electromagnetic power level of a field pattern and the efficiency, and between the RSSI and the power.

(1) $$G=\eta_{cd}\left(1-\left|\Gamma^{2}\right|\right)D.$$

(2) $$\mathrm{RSSI}=\frac{P_{Rad.}{G\eta }_{Ant.}}{P_{PathLoss}}.$$

In other words, the antenna gain as the product of directivity and efficiency is put behind the efficiency, since, if a field pattern is concentrated at an undesirable angle as in back-radiation, although the gain increases with the directivity, the gain becomes meaningless. For a very small antenna which tends to have lower antenna efficiency compared to bigger geometries with fewer layers, the wide-beam pattern in the boresight and the efficiency should be checked rather than the gain. Considering the efficiency, this antenna revealed a proper function in the free space similar to other practices adopting conventional antennas. To become a wearable device, it should be attached to a human body part and result in no functional exacerbation. However, when the antenna was placed in the head–ear phantom, as shown in Figure 2d, the antenna efficiency worsened by 3%. The human phantom named HEAD-P10 has a relative permittivity of 25.7 and electric conductivity of 1.32 (S/m) at 2.45 GHz. This lossy material with a high relative permittivity mimicking the human head and ears screws up the antenna radiation and impedance matching. In Figure 2e, we can see a severe mismatch in the input impedance. The radiated field became aggravated in the broadside, as shown in Figure 2f. The SAR was evaluated to be 0.119 W/kg with an input power of 9 dBm for a volume of 1 g. Therefore, these shortcomings motivated us to devise a novel antenna robust against changes in the placement conditions, showing good radiation efficiency as a small-volume antenna and little difference between being in free space and being worn by a human body part. The aim was to minimize the fringing field coupling due to the effect of human tissue by reducing the length of the radiating element and to make up for the degradation in impedance matching and radiation of the size-reduced radiator. Considering these necessities and the feasibility of being tucked in the tight space of the earbud, a novel antenna is proposed with the parameters described in Table 1.

**Figure 3:**
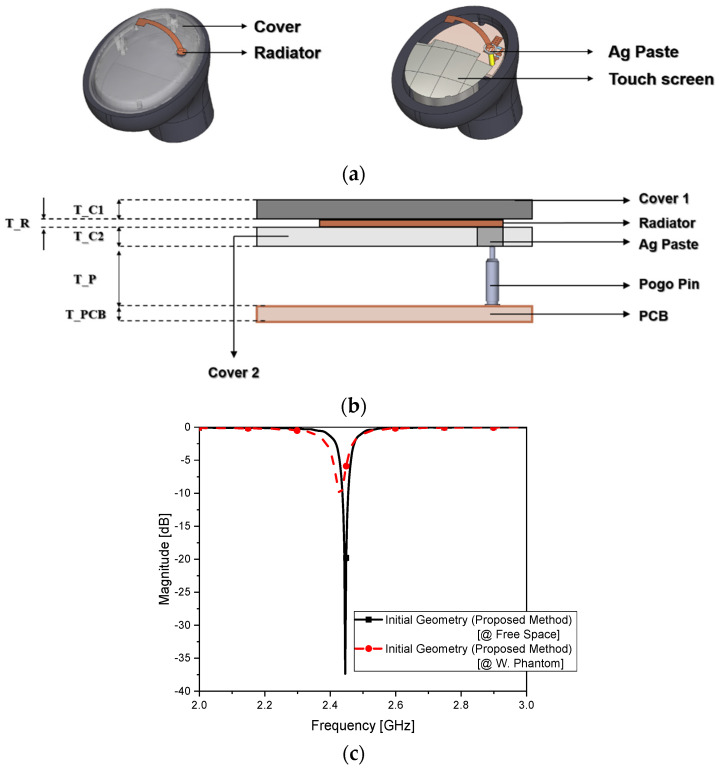

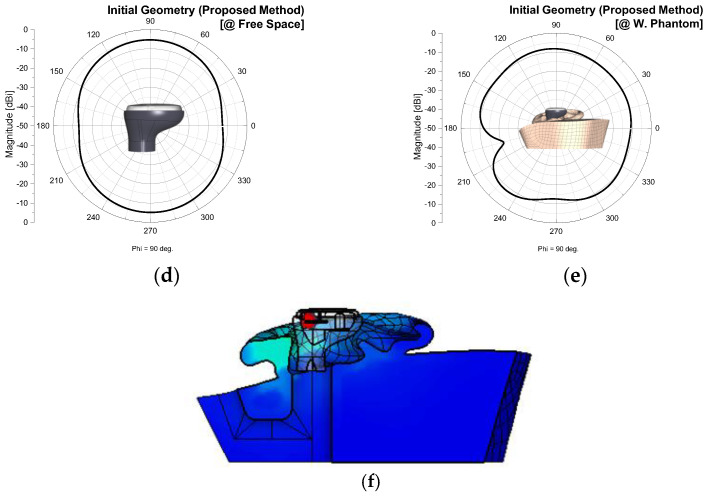
On−film antenna earphone in free space: (**a**) initial geometry of the proposed antenna; (**b**) layer structure; (**c**) S_11_; (**d**) radiated field pattern in free space; (**e**) radiated field attached to the human phantom; (**f**) SAR.

The design of the proposed antenna was broken down into procedural steps, because it was composed of several blocks. The OFAA, shaped as a radiator with approximately one-eighth of the wavelength, was combined with the L-network and was later developed into the final geometry accompanying an AMC. In Figure 3a, the geometry in the initial step is shown as a very short radiating element. This was formed on the film, which is adequate for conforming to the curved surface required by earbud makers. The length of the radiator was 12 mm and featured a copper pattern on a layer sandwiched by cover 1 and cover 2, whose dielectric constant and thickness were 3.2 and 0.7 mm, and 2.9 and 0.55 mm, respectively, as shown in Figure 3b. A radiating element much shorter than a conventional one would worsen the return loss, as the joints from the multiple layers can already sway the state of impedance matching. To iron out the problem, the radiating element was designed cooperatively with the L-network. This led to S_11_ in Figure 3c with parameters of 12 mm, 4.6 nH, and 2.0 pF. Its radiated field pattern is plotted in Figure 3d. The antenna efficiency was 21% in the free space. This was lower than that of the conventional structure, but it remained to be seen whether there were positive points from this short antenna when put in the ear. Figure 3e shows the characteristics of the antenna when positioned in the ear phantom. Similar to the change in Figure 2, the impedance matching was degraded with the frequency shift and increased S_11_, shown in Figure 3c as a frequency shift of 15 MHz and mismatch jump of 27 dB, because of the effect of the human tissue. The efficiency decreased to 10% when loaded in the ear phantom. Despite being much better than the conventional antenna, to ensure that the beam pattern in Figure 3e was stronger than that in Figure 2f, a measure was needed for preventing a rapid drop in the antenna efficiency or even increasing it with the SAM. As the impact of the wireless device on the human tissue was calculated for the conventional structure, the SAR was evaluated for the case of the short radiator. An input power of 9 dBm was applied according to the BLE datasheet, and the 1 g SAR provided 0.06 W/kg, which is below the 1.6 W/kg standard. Confronting the degradation when wearing the device, a robust method was necessary for adequate functioning. A metasurface called an AMC was devised as an additional functional layer harmonious with the given setting of the earbud. Table 2 has the physical dimensions of the AMC.

**Figure 4:**
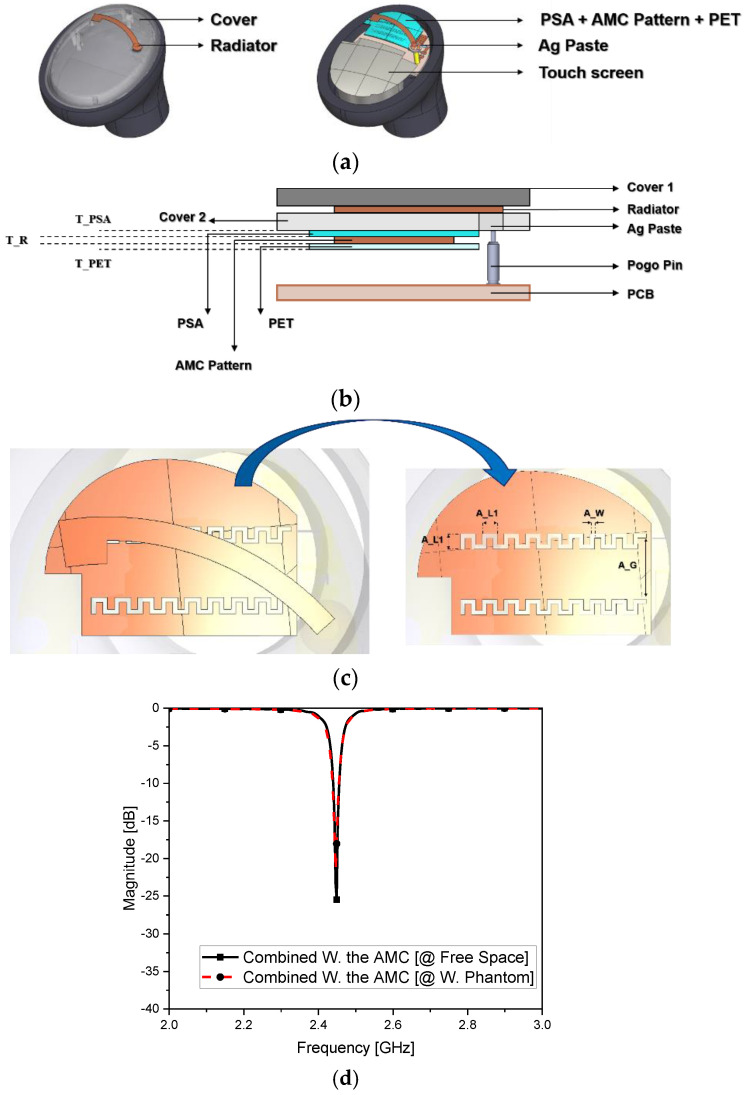

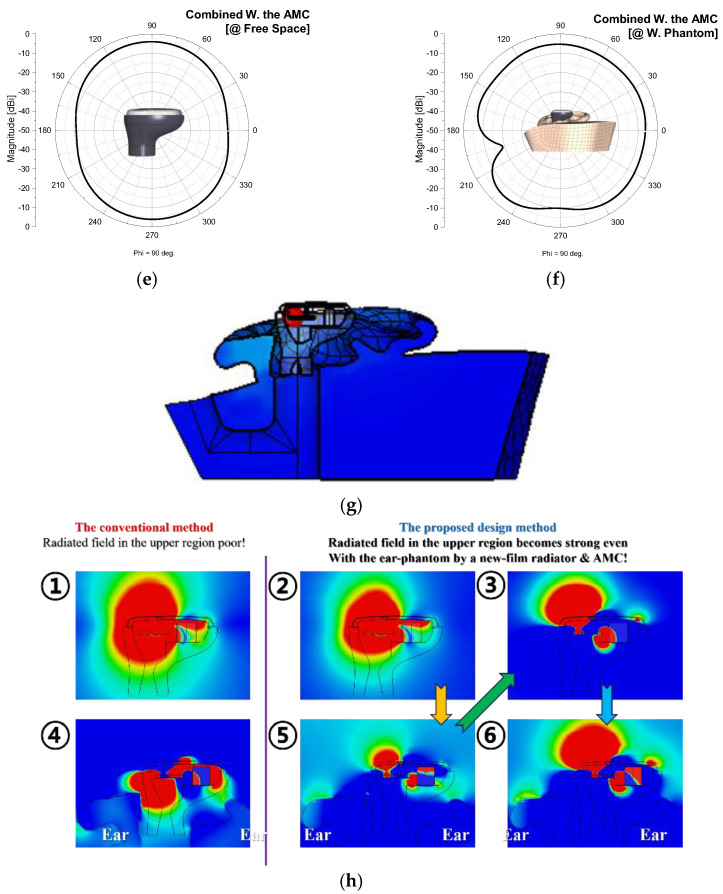
On−film antenna earbud in free space: (**a**) combined with the AMC; (**b**) layer structure; (**c**) geometry of the AMC; (**d**) S_11_; (**e**) radiated field pattern in free space; (**f**) radiated field pattern attached to the human phantom; (**g**) SAR; (**h**) comparison of upward near-field strength.

Considering the small on-film radiating element fed by the impedance-matching circuit, a film AMC structure with a copper pattern was inserted with the purpose of gathering the EM fields upward to the boresight for enhancing the efficiency and mending the error in the lumped-element impedance matching. In Figure 4a, a thin surface is shown to be made of the metal pattern with two corrugated slits on a film. The geometric parameters for the two-pulsated slit metal plane shown in Figure 4b,c allowed avoiding the impedance mismatch and frequency shifting due to excessive capacitive coupling between this new film layer and the already existing radiating film layer, confining the EM fields under the radiating layer. At this point, it is noteworthy that almost no deterioration for this revamped antenna was seen when worn by a human body part. Figure 4d reveals that, without changing the L-network for the antenna, the AMC as a distributed element helped to tune the entire structure at the target frequency. When placed on human tissue, the impedance matching changed with a frequency shift of 3 MHz and a degradation level of 4 dB (outperforming the conventional case). Another positive point of using this metasurface was the improvement in efficiency, reaching 30% with the field pattern shown in Figure 4e. In free space, the small antenna alone performed better than that with the film AMC by a large margin of 9%, whereas it was hard to obtain even a small increase for other very small and complicated antennas. The role of the film AMC was pronounced by the earbud of the OFAA when positioned in the ear phantom. In Figure 4d, this novel device is shown worn by the human phantom. S_11_ presented almost no degradation in the impedance matching from the free space case as in the comparison. This is highly contrary to the conventional antenna and the small antenna just with the lumped-element impedance matching circuits, which were vulnerable to the human tissue effects. However, the proposed antenna enables earbud RF designers to keep the feeding circuit, which is a way to save the cost. The far-field pattern of the proposed antenna in Figure 4e was obtained for the free space. Figure 4f displays the plot of the radiated field pattern of the OFAA loaded in the ear phantom. The antenna loaded on the human tissue revealed no serious impact according to the SAR calculated in Figure 4g (0.032 W/kg) being much lower than the 1.6 W/kg standard limit. A broadside and upward beam was obtained in the boresight, and its efficiency was 22%. This design method could enhance the antenna efficiency of a very small-form factor antenna by approximately 20% and 10% compared to the customary and initially proposed antennas. Moreover, the OFAA secured robustness against the degrading effects of RF equipment when worn on the human body. This was verified by the field plots of all the aforementioned cases shown in Figure 4h. The conventional antenna ended up with poor upward radiation from ① to ④. Nevertheless, regarding the OFAA, the initial geometry started from ② to ⑤ with no AMC, but evolved through ③ to ⑥, thereby accomplishing the desirable upward radiation with a high strength due to the film-type AMC. This advantage was obtained when using the proposed antenna, but not the other metamaterials.

## 3. Electromagnetic Tests of the Antenna Alone and Placed in the Wearable Device

### 3.1. Realization of the OFAA as the Proposed Antenna

The radiator and the AMC of the antenna were fabricated as film structures before being incorporated into the PCB feeding circuit and moved to the inside of the earbud.

**Figure 5:**
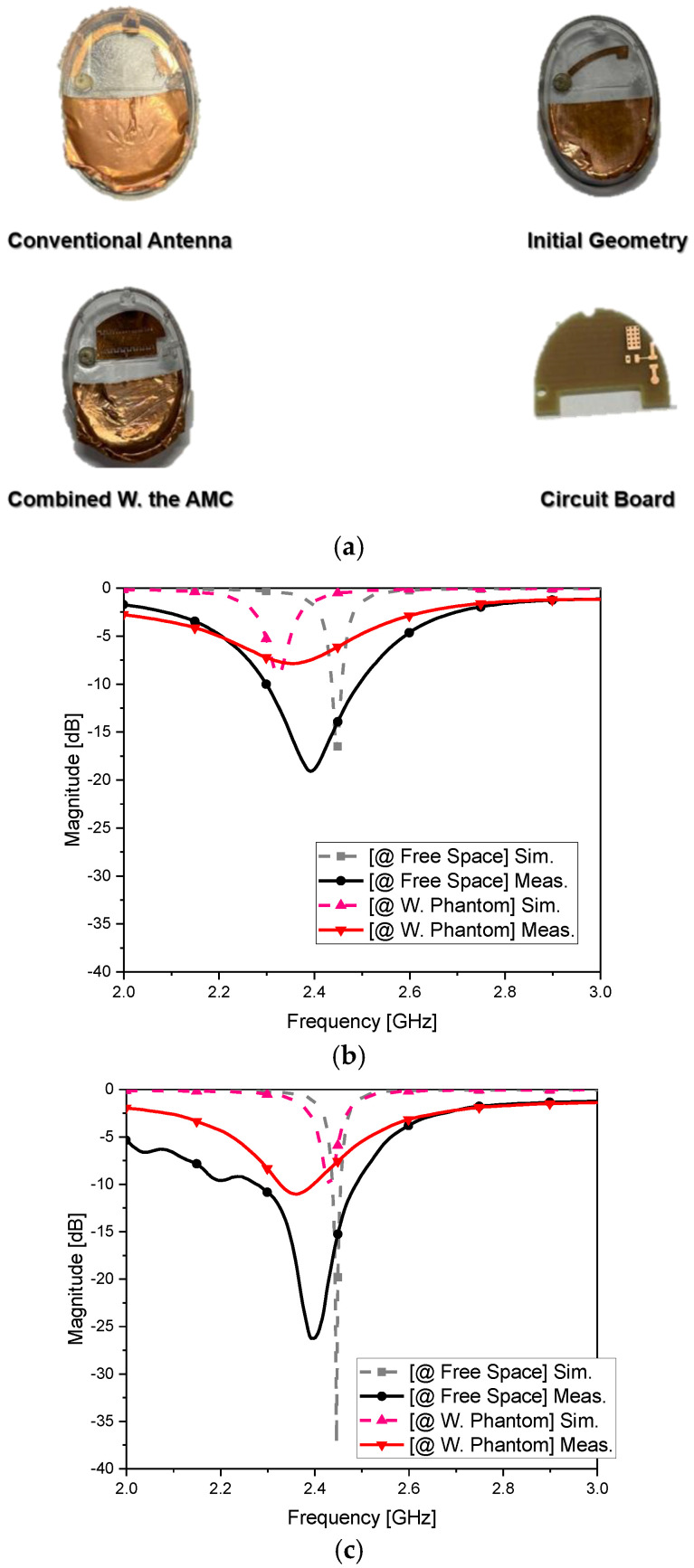

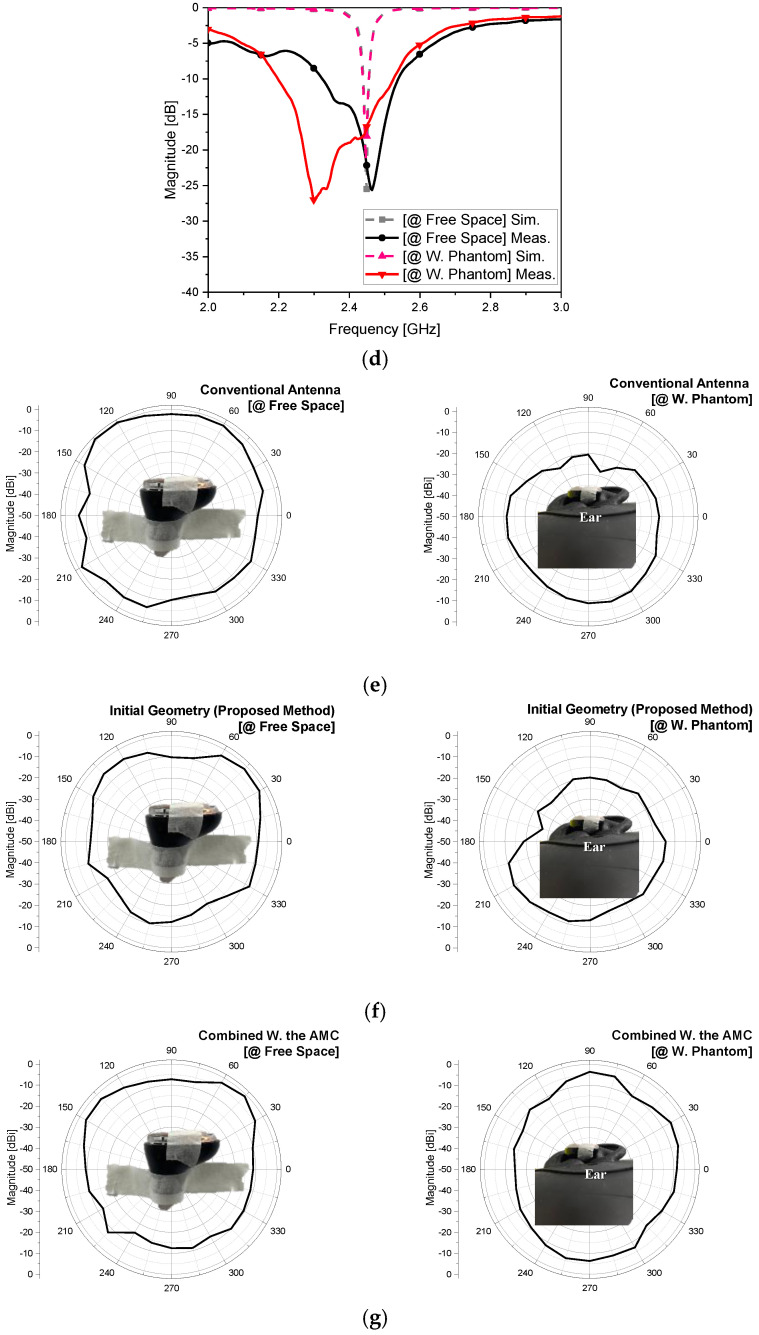
Fabricated antennas and measured S_11_ and far-field patterns for the conventional antenna (CA) and OFAA: (**a**) photographs and S_11_ of (**b**) CA; (**c**) initial geometry (short radiator without the AMC); (**d**) OFAA; (**e**) beam pattern of the CA (**left**) in free space and (**right**) in the ear; (**f**) beam pattern of the initial geometry (**left**) in free space and (**right**) in the ear; (**g**) beampattern of the OFAA (**left**) in free space and (**right**) in the ear.

Figure 5a shows the manufactured film-based radiating elements, long and short radiators, and the film AMC coupled to the short element. Mock-ups were 3D-printed as described in [33]. Figure 5b,c show that the conventional antenna had no problem in terms of the measured S_11_ and far-field pattern in free space, in agreement with the VNA [34]. Similarly, as shown in Figure 5b–d, the initial and final forms of the OFAA exhibited good impedance matching and acceptable radiation in free space. However, the long and short radiators shown in Figure 5b–d presented impedance mismatch and weakened boresight radiation when placed in the ear. The OFAA behaved differently from the other antennas vulnerable to harsh EM disturbance by human tissue. As a reminder, it was backed by another film layer which worked as the AMC to overcome the weaknesses of a tiny antenna, e.g., imperfect impedance matching, lower efficiency, field over-coupling, and excessive field sensitivity. Figure 5a, like Figure 4b,c, displayed the AMC in the middle, possessing two corrugated edged slits of different positions and sizes used to boost the fields into the upper region, as a keynote measure for robustness to the effects of human tissue proximity. This multilayered structure resulted in the S_11_ reflection coefficient at the input port of the feeding circuit shown in Figure 5b,d. Its impedance was matched and acceptable radiation properties (14.9%) were obtained as a very compact antenna. Despite the discrepancy between the simulated and measured S_11_ curves, which resulted from physical implementation with soldering chip-type elements, pin and cable connecting, etc., the reflection coefficient was below −10 dB for impedance matching in the proposed case. The far-field patterns of this OFAA in the EM design step and physical realization showed similar behaviors, suitable for BLE wireless links. Because this antenna is suggested for a wearable device, the observation in free space was further evaluated in human tissue.

### 3.2. Additional Evaluation on the Head–Ear Phantom for the RF Test of the OFAA

The earbud with the film AMC-assisted film antenna was introduced into the hole of an ear phantom attached to the head phantom. POPEYE-V10 is often used as a head–ear phantom [35]. Its CAD model was considered in the EM analysis design step and was tested as something tangible. Its physical properties are described below.

**Figure 6:**
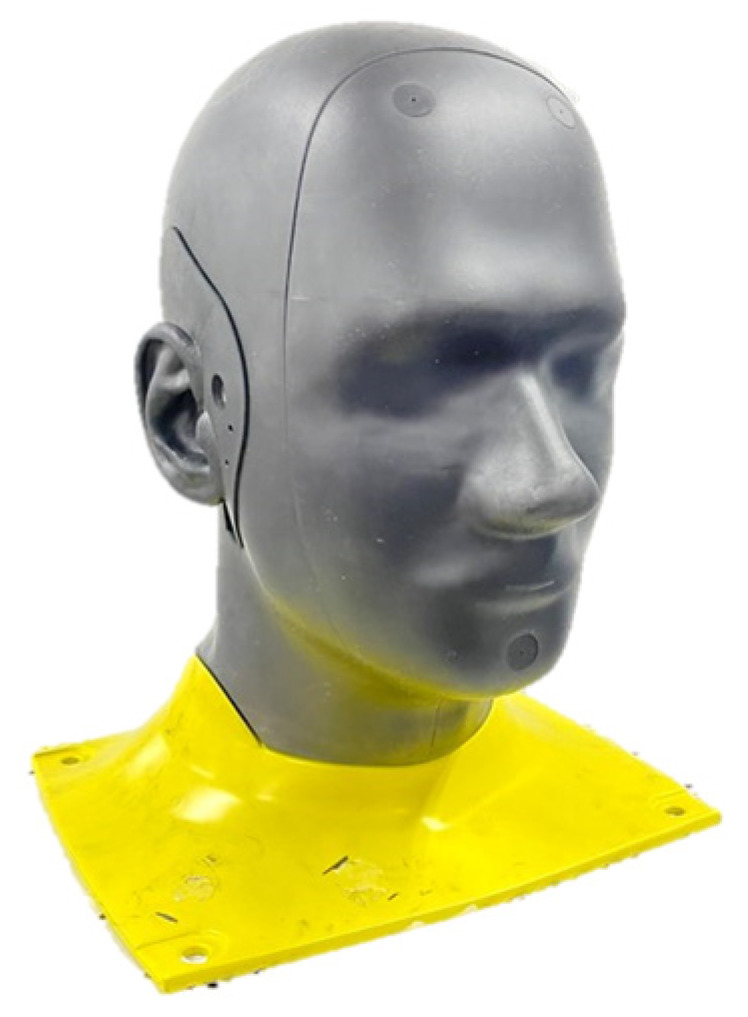
POPEYE-V10 [35].

The human phantom in Figure 6 complies with IEC standards and is a sophisticated model compared to others lacking ears. The ear phantom had a hole in which the proposed antenna system was put. As predicted by the EM simulations in the previous section, the fields from the device were disturbed compared to the free space cases due to the electromagnetic coupling with the nearby human tissue, which resulted in severe degradation in terms of impedance matching and upward radiation. The placement of the device in the earhole surrounded by the highly lossy material worsened the RF properties for ordinary antenna designs. Treated properly, the earbud equipped with the OFAA could diminish the degrading factors with the cooperative interaction between the film-type short radiator and the film-type AMC to guide most of the antenna’s EM fields upward such that they were turned away from the sides in contact with the human tissue. As a positive effect, the resonance frequency remained almost the same as the free space situation according to the S_11_ in Figure 5d. This good impedance matching regarding the earbud-worn human phantom was accompanied by robust radiation performance. The far-field pattern was projected into the observation plane as shown in Figure 5g, producing an adequate radiated field distribution with 14.9% for BLE connectivity. While the efficiency of the conventional antenna from the free space to the human phantom case plunged drastically, the OFAA prevented undesirable deterioration. The fact that the OFAA enabled the earbud in the ear phantom to present recommendable RF characteristics clearly highlights its advantages and superiority compared to the conventional design methods found in quarter-wavelength monopoles, LDS (laser direct structuring), or chip antenna manufacturing techniques. The above-described tests examined the antenna in terms of the electromagnetic aspect; however, it is meaningful to check the wireless link and throughput of the BLE from the communication aspect as a practical method. In particular, communication tests combined with EM power spatial distributions can be applied.

### 3.3. Realistic and New Wireles Test Approach from a Communication System Perspective

Emulating a real-world radio link test aimed at BLE services, the designed antennas were used in a communication-based investigation. The received signal strength indicator (RSSI) test was performed with a module of a chip and an antenna. The far-field antenna test setup (using an RF signal generator and a RF network analyzer) is not appropriate for indicating communication quality because it uses only reference antennas and AUTs, not capable of holding communication modules. To plot the received RF strengths on a pair of distances and angular positions, a new test setup should be built. The electromagnetic power levels were collected and plotted as RSSI distributions at the grid points on the examination area of the spherical surface, representing an in situ test which mimics a real use case with multireflection by the non-free space.

**Figure 7:**
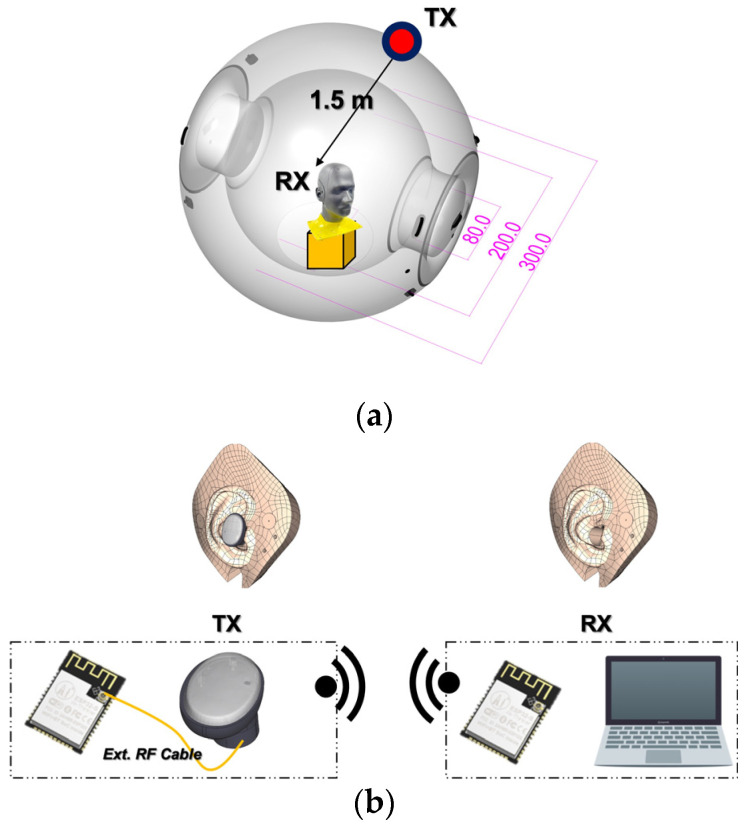

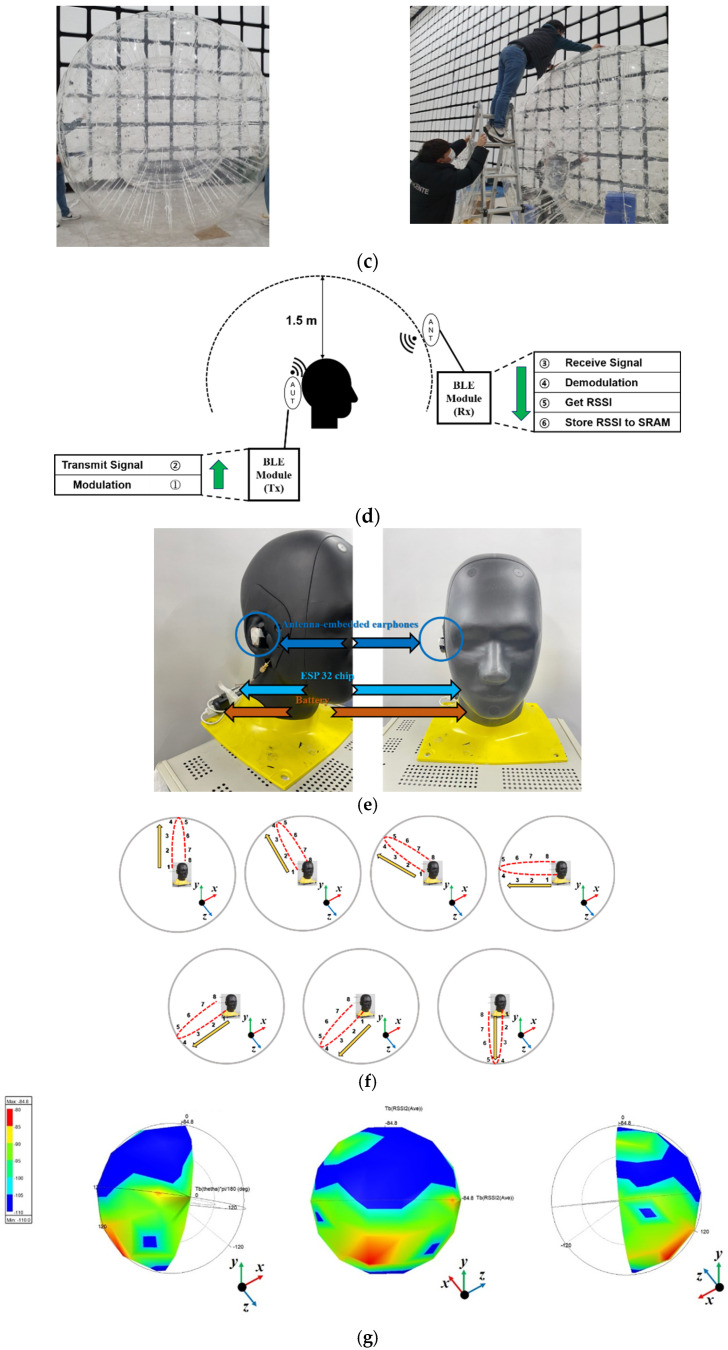

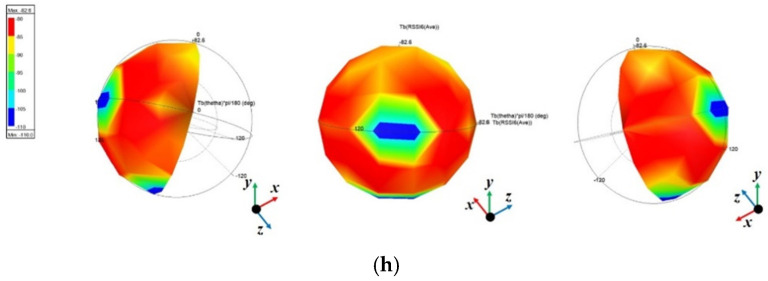
Antennas in the earbuds and RSSI tests using the head phantom: (**a**) plastic zorb ball mimicking a sensing sphere; (**b**) configuration of the measurement; (**c**) human phantom centered in the zorb ball as a TX in the form of a BLE module; (**d**) scheme to collect data from grid of sensors on the sphere; (**e**) device positioned on the SAM for the RSSI test; (**f**) measured points on the latitudes; (**g**) received signal strength distribution plot on the ball about the conventional antenna earbud; (**h**) received signal strength distribution plot on the ball about the proposed antenna earbud.

A plastic ball called a zorb ball was designed and built for the test scenario of the work in this paper. The plastic surface would cause multireflection and scattering. The radius of the ball shown in Figure 7 was 3 m, allowing a person to enter it and facilitating the earbud-equipped head phantom to be placed at its center. Two test cases were evaluated, with the antennas designed using the conventional and proposed methods. In each test case, the head phantom wore the antenna driven by the chip in the BLE module, which worked as the TX. The module used the ESP 32 chip to provide the BLE signal, as shown in Figure 7b. As a note, the communication by the ESP 32 module is bilateral between the TX and RX. The spherical surface was a grid of periodical dents to hold receiver sensors. The indented positions were formed as pairs of azimuth and elevation angles 3 m away from the center of the zorb ball. Figure 7c shows the head phantom wearing the earbud with the embedded antenna. As the receiving sensor, another BLE module was placed on each of the dents as the nodes of the spherical grid, as shown in Figure 7d–f. Not all the positions on the zorb ball were used to prevent time-consuming and repetitive attaching, recording, detaching, and moving of the sensor on the very large ball. The hemispherical sector corresponding to the lit region (line of sight) with reference to the cheek with the earbud was selected. Seven latitudes from the north pole to the south pole were selected with a 26° interval on the sphere. An arc ranging from 0° to 180° on each latitude of the sphere allowed eight equidistant positions for sensing. In total, there were 56 nodes used in each test case. Following repetitive measurements, it took several days to complete data collection in the chartered anechoic chamber, and the 3D maps of the RSSI distribution were constructed for the two cases. The received EM power by the conventional antenna earbud worn by the head phantom presented the RSSI distribution shown in Figure 7g. According to the map, most of the hemisphere was expressed in blue and green, indicating very low power coming from the conventional antenna. The sensor revealed very weak signals with a level of −110 dB to −90 dB in the first test case. The conventional antenna was replaced by the proposed antenna in the second test case, and the RSSI presented a substantial improvement. As shown in Figure 7h, the majority of the 3D map was presented in red, implying that the received signal was strong enough and much stronger than in the first test case. A great enhancement of 20–30 dB was achieved with the use of the OFAA for the BLE earbud.

## 4. Conclusions

To resolve the downsides of a wireless IoT device when operated as a wearable RF sensor, a new antenna with a film-based AMC structure was suggested for BLE earbuds. The conventional antenna has no functional problems in free space, but its performance becomes severely worse when mounted in the human ear, due to degradation in terms of impedance matching and upward field radiation. As the proposed structure named the OFAA, a metal pattern as the short radiator was printed on a film layer probe-fed with a matching circuit in the design of the initial geometry, and another film layer as the AMC was inserted to enable earbuds to prohibit the antenna efficiency and impedance matching due to aggravation, since the suggested antenna was robust against the harsh electromagnetic conditions of human tissue. In electromagnetic analysis of the conventional and proposed antennas, there was a >−30% drop in efficiency from the free space to the in-ear case for the conventional antenna, whereas the OFAA could maintain the antenna efficiency. The OFAA had better performance in terms of the strength of the broadside radiated field, as well as impedance matching, in the wearable situation. These antennas were fabricated and tested using POPEYE-V10 as the human phantom. The measured s-parameters and field patterns revealed the validity of the design method, and the OFAA was superior to the conventional antenna when put in the ear phantom. The conventional antenna had an extremely low efficiency, but the AMC-assisted antenna presented at least 10% greater efficiency than the ordinary antenna. Furthermore, the proposed antenna far-field patterns were stronger than those of the quasi quarter-wavelength antenna when worn by the ear phantom. This advantage became convincing when assessing the RSSI of the BLE module with our new in situ test environment featuring a zorb ball and realistic BLE link tests. The head phantom equipped with the conventional and proposed antennas in turn was located in the center of the zorb ball. The earbud in the head phantom worked as the RX, while the ESP 32 chipset of the BLE module was attached to the dents of the zorb ball as the TX and moved to subsequent points according to the test scenario. The zorb ball mimicked the spherical surface of the field measurement and offered a sensor grid, which resulted in a map of the electromagnetic power strengths. According to the analysis of the RSSI values on the sensor grid, a 20–30 dB enhancement in BLE communication was achieved by the proposed antenna compared to the conventional one. This reveals that the OFAA can play a meaningful role in linking wearable IoT devices in offices and factories.

## Figures and Tables

**Table 1:** The physical dimensions of the initial geometry of the proposed antenna.

Parameter	Value
T_C1	0.700 mm
T_R	0.018 mm
T_C2	0.550 mm
T_P	3.000 mm
T_PCB	0.800 mm

**Table 2:** The physical dimensions of the antenna combined with the AMC.

Parameter	Value
T_PSA	0.05 mm
T_PET	0.10 mm
A_L1	0.7 mm
A_W	0.2 mm
A_G	3.0 mm

## Data Availability

Not applicable.
